# Harnessing the Natural Acidity of Raw Passion Fruit Juice for Pathogen Inactivation in Developing Countries

**DOI:** 10.3390/foods15101799

**Published:** 2026-05-19

**Authors:** Ximena Yepez, Adriana Vanegas-Torres, Hansel A. Mina, Herta Montoya, Manuel Salmeron, Dharmendra K. Mishra, Amanda J. Deering

**Affiliations:** 1 Department of Food Science, Purdue University, 745 Agriculture Mall, West Lafayette, IN 47907, USA; xvyepez@espol.edu.ec (X.Y.); avanega@purdue.edu (A.V.-T.); adeering@purdue.edu (A.J.D.); 2 Escuela Superior Politécnica del Litoral, Facultad de Ingeniería en Mecánica y Ciencias de la Producción, ESPOL, Km 30.5 Vía Perimetral, Guayaquil 90902, Ecuador; 3 Lyles School of Civil Engineering, Purdue University, 585 Purdue Mall, West Lafayette, IN 47907, USA

**Keywords:** passion fruit juice, high-acid matrix, pathogen inactivation, non-thermal processing, food safety, developing countries

## Abstract

Unpasteurized fruit juices in developing countries pose significant public health risks due to potential contamination with foodborne pathogens, particularly in rural areas where reliable energy for thermal processing is lacking. This study evaluates the natural acidity of passion fruit juice as a non-thermal strategy to inactivate *Salmonella ser.* Typhimurium, *Escherichia coli* O157:H7, and *Listeria monocytogenes*. Pathogens were inoculated into passion fruit juice at pH 2.9, 3.4, and 3.9, and their survival was monitored at 25 °C (room temperature) and 5 °C (refrigerated). Log-linear and Weibull models were used to predict inactivation kinetics, targeting a 5-log reduction in accordance with FDA requirements. At pH 2.9 and 5 °C, *S.* Typhimurium and *E. coli* achieved a 5-log reduction within 8 h, while *L. monocytogenes* required 24 h to achieve the same reduction level. The Weibull model provided a superior fit (R^2^ > 0.94) at pH 2.9 and 3.4, accurately capturing the nonlinear inactivation dynamics. Increasing pH to 3.9 significantly slowed inactivation, underscoring the critical role of low pH. These findings suggest that the inherent acidity of passion fruit juice provides a practical, energy-independent method for controlling pathogenic bacteria in developing regions, preserving nutritional quality without thermal processing.

## 1. Introduction

The global demand for tropical fruit juices, such as passion fruit (*Passiflora edulis*), is rising due to their unique flavors and nutritional benefits [1]. In developing countries, where passion fruit is often cultivated in remote areas, producing safe, value-added products such as juices is crucial for economic growth and food security. However, unreliable access to electricity in these regions limits the use of thermal processing, increasing the risk of contamination by foodborne pathogens [2,3]. The presence of human pathogens in juice can be attributed to contamination of fruits and vegetables during preharvest practices, such as irrigation with contaminated water or direct contact of the produce with agricultural soil that has often been amended with manure [3,4]. Additionally, fruits and vegetables can become contaminated in postharvest facilities through the use of non-potable water for washing processes or through poor sanitation practices that lead to cross-contamination [5,6,7]. Under the right conditions, these food products provide a nutrient-rich environment that supports the survival and proliferation of bacteria throughout the entire production process [8,9]. The Centers for Disease Control and Prevention (CDC) has reported numerous foodborne outbreaks associated with fruit juices. Most of these outbreaks are linked to the consumption of unpasteurized products [10]. Notable outbreaks include *E. coli* O157:H7 in Kansas linked to the consumption of unpasteurized apple cider [11], a multistate outbreak of *Salmonella* Typhimurium and *Salmonella* Saintpaul from unpasteurized orange juice [12], and a *Salmonella* Typhi outbreak in 2010 from an imported frozen mamey fruit pulp from Central or South America [13].

Although pathogenic bacteria such as *Salmonella* Typhimurium, *E. coli* O157:H7, and *Listeria monocytogenes* typically do not grow at pH levels below 4.5, their survival in acidic environments varies [14,15]. The U.S. Food and Drug Administration (FDA) mandates a 5-log reduction in the most resistant pathogen in fruit juices to ensure safety throughout shelf life [16]. Passion fruit juice, with a natural pH of 2.7–3.2, may harness its high acidity to achieve this standard without thermal treatment, which can degrade vitamins, flavor, and bioactive compounds [17]. For instance, orange juice at pH 3.5 achieved a 6-log reduction in *Salmonella* after 14 days of refrigeration, compared to 43 days at pH 4.0 [18], suggesting the role of acidity in pathogen control.

In tropical regions, such as South American rainforests, passion fruit processing facilities often lack the infrastructure for pasteurization, driving demand for “natural” frozen juices that preserve thermolabile nutrients and flavor. This study serves as a proof of concept for using the inherent acidity of passion fruit juice as a non-thermal pathogen control strategy, evaluating inactivation kinetics of *S.* Typhimurium, *E. coli* O157:H7, and *L. monocytogenes* at pH levels of 2.9, 3.4, and 3.9 under room temperature (25 °C) and refrigerated (5 °C) conditions. Predictive models (log-linear and Weibull) were developed to estimate inactivation parameters, offering a scalable, energy-independent solution for safe juice production in resource-constrained developing countries.

## 2. Materials and Methods

### 2.1. Bacterial Strain and Inoculum Preparation

Laboratory strains of *Salmonella enterica* serovar Typhimurium ATCC 14028; *E. coli* O157:H7 B6-914 ATCC 43888 (expressing type 1 fimbriae, extracellular polysaccharides, and flagella, which promote bacterial attachment); and *Listeria monocytogenes* 10403S (expressing flagella, internalin, and internalin-like genes, facilitating adhesion) [4] were obtained from the Department of Food Science, Purdue University. These bacteria are handled in a Biosafety Level 2 (BSL-2) laboratory after approval by the Institutional Biosafety Committee (IBC; protocol number 13-006-25). Bacterial strains were streak-plated onto selective media, including Xylose-Lysine-Tergitol 4 (XLT4) for *S.* Typhimurium, MacConkey Sorbitol for *E. coli* O157:H7, and Oxford Medium for *L. monocytogenes*, respectively. The plates were incubated at 37 °C for 24 h. Isolated colonies were grown in Luria–Bertani broth for *S.* Typhimurium and *E. coli* O157:H7, while Brain Heart Infusion broth was used to grow *L. monocytogenes*. Cultures were incubated at 37 °C and 150 rpm for 18 h. Then, bacterial cultures were washed by centrifugation at 4500× *g* for 5 min. Bacterial pellets were resuspended to a final cell density of 10^8^ CFU/mL, using 30 mL of 0.1 M phosphate buffer pH 7.0 (PB). This washing procedure was repeated a total of three times. Upon resuspension, the optical density (OD_600_) was measured using a HACH-meter DR 2800 spectrophotometer (HACH, Loveland, CO, USA) at 600 nm [4]. Each strain was independently inoculated into passion fruit juice for subsequent experiments.

### 2.2. Juice Inoculation

Frozen pasteurized passion fruit juice (Les Vergers Boiron, France) was purchased from Webstaurant Store (Pennsylvania, USA) and stored at −18 °C until use. Samples were thawed overnight at 5 °C. For inoculation, 18 mL of juice was mixed with 2 mL of fresh bacterial culture (1:10 ratio) in 50 mL centrifuge tubes, then vortexed thoroughly. Each pathogen (*S.* Typhimurium, *E. coli* O157:H7, and *L. monocytogenes*) was inoculated independently. Samples corresponding to a specific pH level were prepared, stored, and handled separately. Inoculated samples were stored at 25 °C (room temperature) or 5 °C (refrigerated) until analysis. Independent destructive samples were analyzed at each time point. At designated time points (0–96 h), samples were serially diluted in phosphate-buffered solution (PB) and plated in triplicate on XLT4 for *S.* Typhimurium, MacConkey Sorbitol for *E. coli* O157:H7, and Oxford Medium for *L. monocytogenes*. Plates were incubated at 37 °C for 36 h, and colonies were enumerated to determine the bacterial population.

### 2.3. Physicochemical Characterization

The initial pH of the passion fruit juice was 2.9, and it was adjusted to 3.4 or 3.9 using 2 N NaOH and measured with a calibrated digital pH meter (Fisherbrand FE150, Thermo Fisher, Waltham, MA, USA). Total soluble solids (°Brix) were determined using a digital refractometer (LR-01, Maselli, Bologna, Italy). Titratable acidity was quantified using the AOAC method: 20 mL of juice was diluted with 50 mL of distilled water and titrated against 1 N NaOH to a pH endpoint of 8.2 ± 0.1. The volume of NaOH consumed was used to calculate citric acid content (g/100 mL of juice).

Passion fruit juice was centrifuged at 6000 rpm for 5 min; the supernatant was diluted 1:10,000 times and filtered through a 0.45 μm PTFE filter syringe. Individual organic acid standards were used for a calibration curve (0.025–2 mg/mL). Measurements were performed with 3 replicates. Organic acid content was quantified with an Agilent 6545 UPLC/quadrupole time-of-flight mass spectrometer (Palo Alto, CA, USA) fitted with a T3 column (1.8 μm particle size, 2.1 mm × 100 mm), injection volume of 5 μL and mobile phase (0.1% formic acid) with a flow rate of 0.3 mL/min. A negative mode electrospray ionization (ESI) was used in the mass spectrometer, and high mass accuracy spectra were collected between 80 and 1100 *m*/*z*.

### 2.4. Mathematical Modeling of Inactivation Kinetics

Two models were evaluated to describe the kinetics of bacterial inactivation in inoculated juice over time. The first is the log-linear model, given by the following:

(1) $${log}_{10}N\left(t\right)={log}_{10}N_{0}-\frac{t}{D}$$

where log_10_ N(t) is the logarithm of the microbial population at time t, and log. N0 is the logarithm of the initial population. D is the decimal reduction time, a parameter that provides the time (h) required to reduce 1 log of CFU/mL under the conditions exposed by passion fruit juice acidity. During the inactivation process, a base-10 scale is more suitable than a base-e scale for describing several orders of magnitude. Thus, a base-10 system was used for mathematical modeling [19]. The linear equation was solved using the “fitlm” function in MATLAB (R2023b v23.2, The MathWorks Inc., Natick, MA, USA). The initial cell density (Log N0) and decimal reduction time (D) were estimated using a one-step regression method in the linear model. For the second model, a nonlinear bacterial growth derived from the Weibull distribution function was used [20], given by the following:

(2) $${log}_{10}\left[S\left(t\right)\right]=-b\times t^{n}$$

where S(t) is the survival ratio of N(t)/N0, being N(t) the microbial population at time t and N0 the initial microbial population. The scale (b) and shape (n) parameters were estimated deterministically from the experimental data set. The nonlinear differential equation was solved numerically with the “ode45” function in MATLAB (The MathWorks Inc., 2023). The accuracy of the models was evaluated using root mean squared error (RMSE), sum of squared errors (SSE), Akaike’s information criterion (AIC), and coefficient of determination (R^2^).

### 2.5. Experimental Design and Statistical Analysis

A factorial design was used to evaluate the log reduction in *S.* Typhimurium, *E. coli* O157:H7, and *L. monocytogenes* in passion fruit juice under two storage conditions: room temperature (25 °C) and refrigeration (5 °C), across three pH levels (2.9, 3.4, and 3.9). Each pathogen was inoculated independently, and data were analyzed separately. At 25 °C, samples were assessed at three time points (0, 8, and 24 h), while at 5 °C, eight time points were evaluated (0, 4, 8, 12, 24, 48, 72, and 96 h). All treatments were performed in triplicate. Bacterial populations were expressed as log CFU/mL of juice. One-way ANOVA was conducted to assess the effects of pH and storage time on pathogen inactivation for each bacterium, followed by Tukey’s post hoc test for pairwise comparisons (*p* < 0.05). Statistical analyses were performed using JMP Pro v16 (JMP Institute Inc., Cary, NC, USA), with means and standard errors calculated for all measurements.

## 3. Results

### 3.1. Physicochemical Properties

The samples of passion fruit juice used for room temperature (25 °C) experiments had a pH of 2.9, a citric acid content of 3.7 g/100 mL, and a total soluble solids content of 12.6 °Brix. For refrigeration (5 °C) experiments, juice pH was adjusted to 2.9, 3.4, or 3.9. Titratable acidity and soluble solids content are presented in Table 1. No significant differences in soluble solids content were observed across pH levels. Passion fruit juice is highly acidic, with citric acid as the predominant organic acid. The spectra for citric, ascorbic, and malic acid were identified with the exact masses of 191.0197, 175.0248, and 133.0142 *m*/*z* in the negative mode, respectively. The quantification of organic acids was obtained by a standard curve of each organic acid with concentrations of 0.025–2.000 mg/mL. The identification of organic acids in passion fruit juice (pH 2.9) included citric and malic acid, with a content of 272.6 ± 14.2 mg/mL and 79.2 ± 6 mg/mL, respectively.

### 3.2. Effect of Room Temperature Storage (25 °C)

**Figure 1:**
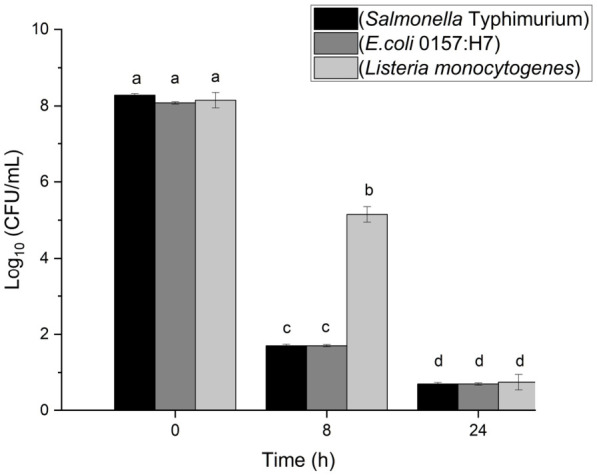
Microbial enumeration of *S.* Typhimurium, *E. coli* O157:H7, and *L. monocytogenes* at room temperature (25 °C), with passionfruit juice with a pH of 2.9. Different letters indicate significant differences between the bacterium groups (*p* < 0.05).

At pH 2.9 and 25 °C, all pathogens exhibited significant reductions in microbial load after 24 h, surpassing the FDA’s 5-log reduction requirement (Figure 1). *S.* Typhimurium, *E. coli* O157:H7, and *L. monocytogenes* populations fell below the detection limit (0.70–0.75 log CFU/mL) by 24 h (*p* < 0.05). The detection limit of the enumeration method was 0.7 log CFU/mL, based on the lowest plated dilution (10^1^). Samples with no detectable colonies were considered below the detection limit and were assigned a value corresponding to 0.5 CFU at this dilution for log transformation and inclusion in model fitting. After 8 h, the Gram-negative pathogens *S.* Typhimurium and *E. coli* O157:H7 showed reductions of 6.6 ± 0.03 and 6.4 ± 0.04 log CFU/mL, respectively. At the same time, the Gram-positive *L. monocytogenes* exhibited a significantly lower reduction of 3.0 ± 0.02 log CFU/mL (*p* < 0.05).

### 3.3. Effect of Refrigerated Temperature Storage (5 °C)

**Figure 2:**
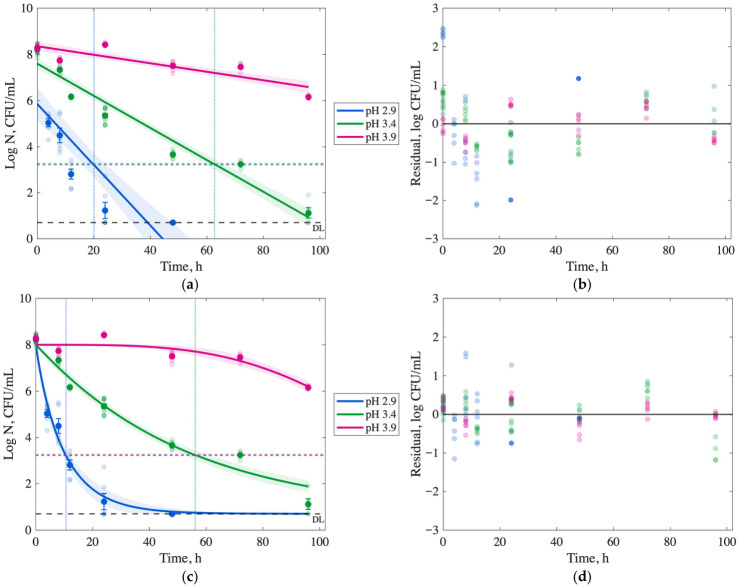
Microbial enumeration of *Salmonella* Typhimurium at refrigeration temperature (5 °C) in passion fruit juice with a pH of 2.9, 3.4, and 3.9. Model fit (solid line) based on average cell density over time. Detection Limit (DL) and 5-log cell density Reduction (LR, dashed line) are illustrated. Vertical lines correspond to the time required to achieve a 5-log reduction. Predicted and experimental log N vs. time (**a**) for the log-linear model and residuals scatter plot (**b**). Predicted and experimental log N vs. time (**c**) for the Weibull model and residuals scatter plot (**d**). For all curves, the blue markers and lines refer to passion fruit juice with pH 2.9, green indicates pH 3.4, and red indicates pH 3.9.

**Figure 3:**
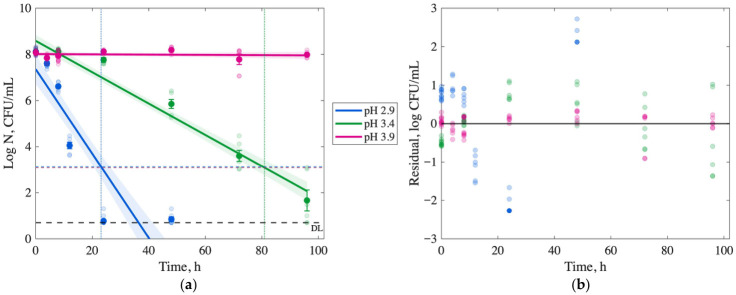

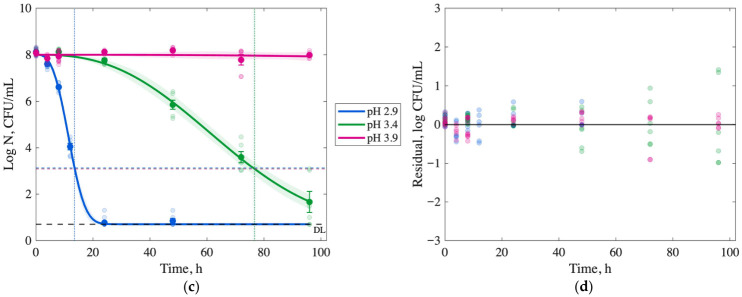
Microbial enumeration of *E. coli* O157:H7 (2) at refrigeration temperature (5 °C), in passion fruit juice with a pH of 2.9, 3.4, and 3.9. Model fit (solid line) based on average cell density over time. Detection Limit (DL) and 5-log cell density Reduction (LR, dashed line) are illustrated. Vertical lines correspond to the time required to achieve a 5-log reduction. Predicted and experimental log N vs. time (**a**) for the log-linear model and residuals scatter plot (**b**). Predicted and experimental log N vs. time (**c**) for the Weibull model and residuals scatter plot (**d**). For all curves, the blue markers and lines refer to passion fruit juice with pH 2.9, green indicates pH 3.4, and red indicates pH 3.9.

**Figure 4:**
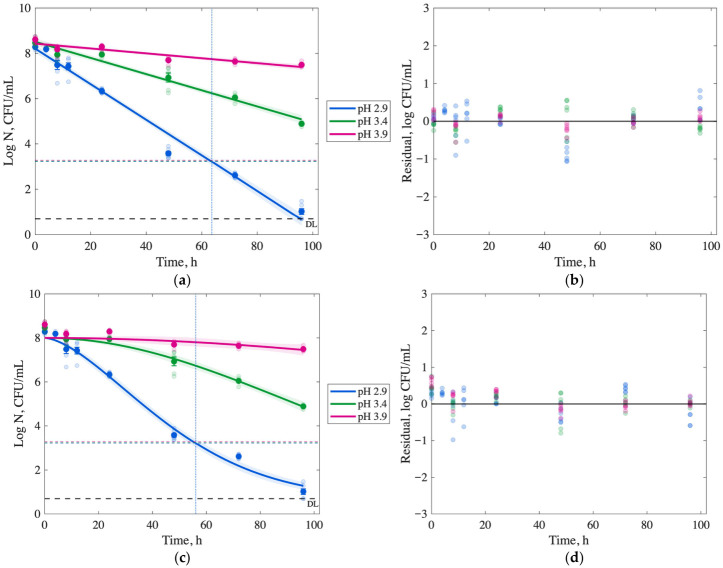
Microbial enumeration of *Listeria monocytogenes* at refrigeration temperature (5 °C) in passion fruit juice with a pH of 2.9, 3.4, and 3.9. Model fit (solid line) based on average cell density over time. Detection Limit (DL) and 5-log cell density Reduction (LR, dashed line) are illustrated. Vertical lines correspond to the time required to achieve a 5-log reduction. Predicted and experimental log N vs. time (**a**) for the log-linear model and residuals scatter plot (**b**). Predicted and experimental log N vs. time (**c**) for the Weibull model and residuals scatter plot (**d**). For all curves, the blue markers and lines refer to passion fruit juice with pH 2.9, green indicates pH 3.4, and red indicates pH 3.9.

Microbial inactivation of *S.* Typhimurium, *E. coli* O157:H7, and *L. monocytogenes* in passion fruit juice at 5 °C across pH levels (2.9, 3.4, and 3.9) is presented in Figure 2, Figure 3 and Figure 4 and Supplementary Materials. The initial microbial populations of the inoculum stocks of *S.* Typhimurium, *E. coli* O157:H7, and *L. monocytogenes* were 8.34 ± 0.29, 8.8 ± 0.01, and 9.21 ± 0.46 log CFU/mL, respectively. At pH 2.9, all pathogens were reduced to <1 log CFU/mL within 24–72 h (*p* < 0.05), with *S.* Typhimurium requiring 48 h, *E. coli* O157:H7 24 h, and *L. monocytogenes* 72 h. Per FDA’s 5-log reduction requirement (indicated by the LR line in Figure 2 and Figure 3), *S.* Typhimurium achieved a 5-log reduction within 12 h at pH 2.9, while *E. coli* O157:H7 and *L. monocytogenes* achieved 4.06 ± 0.13 and 7.42 ± 0.09 log reductions, respectively, at the same time point (*p* < 0.05).

At pH 3.4, microbial reductions were slower but significant after 96 h (*p* < 0.05), with *S.* Typhimurium and *E. coli* O157:H7 achieving 7.1 and 6.4 log reductions, respectively, while *L. monocytogenes* showed a 3.6 log reduction. At a pH of 3.9, inactivation rates were significantly reduced (*p* < 0.05). After 48 h, *S.* Typhimurium and *L. monocytogenes* exhibited minimal reductions of 0.8–0.9 log CFU/mL, and *E. coli* O157:H7 showed no significant reduction after 96 h (*p* > 0.05), indicating limited inactivation at higher pH levels.

### 3.4. Modeling Inactivation Curves

Survival curves for *S.* Typhimurium, *E. coli* O157:H7, and *L. monocytogenes* in passion fruit juice at 5 °C across pH levels (2.9, 3.4, and 3.9) are presented in Figure 2, Figure 3 and Figure 4 as log CFU/mL versus time (h). Log-linear and Weibull models were fitted to predict the time required for a 5-log reduction, as required by FDA regulations, with model fits shown in subplots (a) and (c), respectively, and corresponding residual plots in (b) and (d). The limit of detection (DL, 0.70–0.75 log CFU/mL) and 5-log reduction threshold (LR) are indicated in the graphs with horizontal lines. Model parameters and fitness metrics (R^2^, RMSE, SSE, AIC, and BIC) are summarized in Table 2 and Table 3.

At pH 2.9, the Weibull model predicted 5-log reductions within 10.6 h for *S.* Typhimurium, 13.5 h for *E. coli* O157:H7, and 56 h for *L. monocytogenes* (Table 3), outperforming the log-linear model with a higher R^2^ (>0.95) and lower RMSE (<0.6 log CFU/mL), SSE, and AIC values. Figure 2, Figure 3 and Figure 4 show the 5-log reductions with a vertical line in (a) and (c) figures. The Weibull model effectively captured non-linear inactivation kinetics, with residual plots showing minimal deviation from the identity line compared to the log-linear model. For *S.* Typhimurium and *E. coli* O157:H7 at pH 2.9 and 3.4, the Weibull model yielded R^2^ values of 0.95–0.99, indicating a robust fit. For *L. monocytogenes* at pH 2.9, the Weibull model yielded an R^2^ of 0.98, an RMSE of 0.36, and an AIC of –96.14, indicating a smooth reduction to the detection limit.

At higher pH levels (3.4 and 3.9), the log-linear model provided a better description of *L. monocytogenes* inactivation compared to the Weibull model, with R^2^ values of 0.95 (pH 3.4) and 0.80 (pH 3.9). However, it predicted slower inactivation, requiring 28 h and 92.7 h for a 1-log reduction, respectively, and failing to achieve a 5-log reduction within 96 h. Both models exhibited a poor fit at pH 3.9 for all pathogens due to limited inactivation, underscoring the critical role of low pH in controlling pathogens effectively.

## 4. Discussion

### 4.1. Physicochemical Properties

The natural pH of commercial passion fruit juice typically ranges from 2.7 to 3.2, as observed in varieties such as *Passiflora edulis* Sims and *P. edulis f. flavicarpa* Degener [21,22]. However, certain varieties, such as *Passiflora caerulea*, can exhibit a higher pH, up to 3.9 [23]. To capture this variability and assess its impact on pathogen inactivation, this study evaluated juice at pH levels of 2.9, 3.4, and 3.9. The observed decrease in titratable acidity (from 3.72 to 2.40 g citric acid/100 mL) with increasing pH (Table 1) resulted from the addition of NaOH, which neutralizes hydronium ions (H_3_O^+^) and organic acid-bound hydrogen ions. This pH-dependent acidity is crucial for non-thermal pathogen control, as lower pH enhances microbial inactivation, providing a practical solution to ensure juice safety in developing countries where thermal processing is often infeasible.

The juice used in this study had low ascorbic acid content, which may be attributed to losses from thermal treatment. According to Janzantti et al., fresh pulp of passion fruit has 11.4 mg of ascorbic acid/100 mL [24]. It was assumed that the organic acid content of passion fruit juice remained constant across samples with adjusted pH.

### 4.2. Effect of Room Temperature Storage (25 °C)

The low pH of passion fruit juice (2.9) creates an acidic environment that can inhibit the survival of both Gram-positive and Gram-negative bacteria at 25 °C. High proton concentrations induce acid stress, disrupting cytoplasmic pH homeostasis, genetic material stability, and enzyme function [25]. Consequently, *S.* Typhimurium and *E. coli* O157:H7 exhibited rapid inactivation, achieving > 6 log reductions within 8 h, while *L. monocytogenes* showed greater resistance, with a 3-log reduction at 8 h and a 5-log reduction by 24 h (Figure 1). This aligns with reports that *L. monocytogenes* employs robust acid stress response mechanisms, including enhanced detection and activation of protective cellular functions [25]. The rapid inactivation of pathogens at pH 2.9 supports the potential of passion fruit juice’s natural acidity as an effective non-thermal control strategy, particularly in developing countries where ambient storage is common due to limited refrigeration.

### 4.3. Effect of Refrigerated Temperature Storage (5 °C)

At 5 °C, *L. monocytogenes* exhibited greater acid resistance compared to *S.* Typhimurium and *E. coli* O157:H7, with slower microbial reductions across a pH range of 2.9–3.9 (Figure 2, Figure 3 and Figure 4). At pH 2.9, *S.* Typhimurium and *E. coli* O157:H7 achieved 5-log reductions within 12–14 h, while *L. monocytogenes* required 60 h, consistent with its robust acid stress response mechanisms [25,26]. Low pH enhances microbial inactivation by organic acids, such as those in passion fruit juice, which penetrate bacterial membranes due to their lipophilic properties, releasing protons that acidify the cytoplasm and disrupt cellular functions [26,27].

Comparative studies support these findings. For instance, *L. monocytogenes* in avocado pulp (pH 6.7) and processed guacamole (pH 5.3) showed 2- and 3-log reductions, respectively, after 58 weeks at −18 °C [28]. Similarly, *L. monocytogenes* in ground beef (pH 5.8) exhibited no significant reduction, while tomato soup (pH 4.7) achieved 4–6 log reductions after 8–14 weeks at −20 °C [29]. These studies highlight the crucial role of low pH in microbial inactivation, with passion fruit juice’s pH of 2.9 facilitating faster reductions than higher-pH matrices, particularly under refrigeration conditions.

The results emphasize the importance of maintaining a low pH (≤3.4) to meet the FDA’s 5-log reduction requirement for juice safety [17]. At pH 3.4, *L. monocytogenes* achieved only a 3.6-log reduction after 96 h, indicating insufficient inactivation to meet compliance requirements within practical storage times. At pH 3.9, inactivation was minimal for all pathogens, underscoring the pivotal role of pH in food safety. These findings provide a proof-of-concept for leveraging the natural acidity of passion fruit juice as a non-thermal control strategy in developing countries where refrigeration may be unavailable. Still, the pasteurization infrastructure is limited [30]. Future research should explore scalable methods to standardize pH (e.g., by adding citric acid) and complementary non-thermal technologies to ensure safety while preserving nutritional and sensory qualities in resource-constrained settings.

### 4.4. Modeling Inactivation Kinetics

Log-linear and Weibull models are widely used to describe microbial inactivation kinetics and predict the time required for a 5-log reduction, traditionally in thermal processing studies [20]. In this study, these models were applied to assess non-thermal inactivation in passion fruit juice at 5 °C across pH levels (2.9, 3.4, and 3.9), providing insights into pathogen behavior under acidic stress. According to the AIC and BIC values, the Weibull model was preferred over the linear model for all treatments, except for *Listeria* at pH levels of 3.4 and 3.9.

Results showed that the Weibull model for *E. coli* at pH 2.9 (Figure 3) exhibits a downward concavity, or a “small shoulder”, at the beginning of the curve (0–4 h), which is not observed in the other pathogens’ curves. This behavior may be a response to the new environment, such as a stress response. Then, the curve is gradually reduced until the inflection point at 20 h, where an upward concavity is observed. The shape of this curve is defined by the parameter *n*, which was fixed to >2.6 for *E. coli*, much higher than the values for the other pathogens. The presence of a “small shoulder” has also been reported by Corradini and Peleg during a slow heat process [31]. The researchers showed that a lower heating rate (0.15 °C/min) applied to *E. coli* results in an extended shoulder compared to a higher rate (1.64 °C/min). An extended shoulder may mean it takes longer for the cells to experience significant inactivation due to a slower buildup of the high-acid stress response. Moreover, the shoulder is visible after a longer time at pH 3.4, with an inflection point at 24 h. In contrast, *S.* Typhimurium (*n* = 1) exhibited no shoulder, with rapid inactivation at pH 2.9 (12 h for 5-log reduction), suggesting greater susceptibility to high acidity. *L. monocytogenes* (*n* = 1.5) showed a similar curve shape but a shallower slope, reflecting its robust acid stress response [25], requiring 60 h for a 5-log reduction at pH 2.9. This study is limited to laboratory-scale kinetic investigations of pathogen inactivation in passion fruit juice and future work should include an applied or field-scale validation.

## 5. Conclusions

This study serves as a proof-of-concept for leveraging the inherent acidity of passion fruit juice (pH 2.9, 3.4, and 3.9) as a non-thermal strategy to control foodborne pathogens, offering a viable solution for developing countries where high costs and unreliable electricity limit thermal processing. At 25 °C and pH 2.9, *S.* Typhimurium and *E. coli* O157:H7 achieved >6-log reductions within 8 h, while *L. monocytogenes* reached a 5-log reduction by 24 h. At 5 °C and pH 2.9, *S.* Typhimurium and *E. coli* O157:H7 attained 5-log reductions within 12–14 h, whereas *L. monocytogenes* required 60 h, reflecting its greater acid tolerance and increasing pH to 3.4 or 3.9 significantly reduced inactivation rates, with *L. monocytogenes* achieving only a 3.6-log reduction after 96 h at pH 3.4 and negligible reductions at pH 3.9, highlighting the critical role of low pH in meeting the FDA’s 5-log reduction requirement.

The Weibull model accurately described inactivation kinetics at pH 2.9 and 3.4 for *S.* Typhimurium and *E. coli* O157:H7 (R^2^ > 0.95, RMSE < 0.6 log CFU/mL) and at pH 2.9 for *L. monocytogenes* (R^2^ = 0.98), capturing non-linear dynamics such as shoulder phases and inflection points (Table 2 and Table 3). In contrast, the log-linear model was less effective, as it does not account for adaptation periods. These findings demonstrate that passion fruit juice’s natural acidity can ensure microbial safety without thermal processing. To optimize this approach, a validated maximum pH threshold (e.g., via citric acid standardization) is essential. This study elucidates the behavior of pathogens in high-acid matrices through a controlled, laboratory-scale kinetic evaluation. Future work will address pilot-scale production, as well as sensory and nutritional analysis, natural contamination conditions, and field validation, to support the development of scalable, energy-independent strategies to enhance juice safety in infrastructure-constrained settings.

## Figures and Tables

**Table 1:** Physicochemical properties of passion fruit juice with adjusted pH.

Samples	Soluble Solids (°Brix)	Titratable Acidity (g of Citric Acid/100 mL)
pH 2.9	12.56 ± 0.12 ^a^	3.72 ± 0.06 ^a,^*
pH 3.4	12.39 ± 0.12 ^a^	3.03 ± 0.06 ^b^
pH 3.9	12.14 ± 0.12 ^a^	2.40 ± 0.07 ^c^

* Different letters within a column are significantly different (*p* < 0.05).

**Table 2:** Log-linear model parameters, error, and fitness metrics for microbial prediction.

		Parameters		Error and Fitness Metrics					
		Log N_0_	D (h)	TLR * (h)	RMSE	SSE	AIC	BIC	Model R^2^
*Salmonella* Typhimurium	pH 2.9	5.86 ± 0.35	7.58 ± 0.89	20.08 ± 1.97	1.47	77.44	31.57	34.74	0.68
	pH 3.4	7.60 ± 0.12	14.39 ± 0.57	62.75 ± 1.78	0.61	19.64	−48.62	−44.68	0.93
	pH 3.9	8.35 ± 0.11	54.45 ± 5.93	NaN	0.40	5.79	−61.78	−58.61	0.71
*E. coli* O157:H7	pH 2.9	7.36 ± 0.32	5.47 ± 0.44	23.11 ± 1.40	1.54	113.81	45.44	49.18	0.77
	pH 3.4	8.58 ± 0.13	14.73 ± 0.60	80.93 ± 2.41	0.63	19.14	−40.14	−36.40	0.93
	pH 3.9	8.01 ± 0.05	1739.18 ± 3212	NaN	0.26	3.49	−143.92	−139.94	0.01
*Listeria monocytogenes*	pH 2.9	8.21 ± 0.09	12.73 ± 0.31	63.64 ± 1.09	0.43	8.68	−78.07	−74.32	0.97
	pH 3.4	8.51 ± 0.08	28.01 ± 1.15	NaN	0.30	3.10	−84.31	−81.14	0.95
	pH 3.9	8.43 ± 0.05	92.67 ± 7.99	NaN	0.19	1.25	−116.93	−113.76	0.80

* TLR (time to reach 5-log cell density reduction), NaN not a number.

**Table 3:** Weibull model parameters, error and fitness metrics for microbial prediction.

		Parameters		Error and Fitness Metrics					
		b	*n*	TLR * (h)	RMSE	SSE	AIC	BIC	Model R^2^
*Salmonella* Typhimurium	pH 2.9	0.051 ± 0.012	0.93 ± 0.10	10.64 ± 0.64	0.60	12.83	−33.14	−29.98	0.95
	pH 3.4	0.008 ± 0.002	1.02 ± 0.05	56.15 ± 2.17	0.46	11.04	79.13	−75.19	0.96
	pH 3.9	8.1 × 10^−9^ ± 2.3 × 10^−8^	3.63 ± 0.63	NaN	0.31	3.47	−80.22	−77.06	0.83
*E. coli* O157:H7	pH 2.9	1.9 × 10^−4^ ± 8.4 × 10^−5^	3.00 ± 0.18	13.54 ± 0.22	0.23	2.541	−137.05	−1333.30	0.99
	pH 3.4	5.6 × 10^−6^ ± 4.6 × 10^−6^	2.62 ± 0.19	76.70 ± 1.55	0.46	10.05	−71.06	−67.31	0.96
	pH 3.9	1.9 × 10^−8^ ± 8.0 × 10^−7^	2.70 ± 9.61	NaN	0.26	3.48	−144.14	−140.16	0.01
*Listeria monocytogenes*	pH 2.9	7.5 × 10^−4^ ± 2.2 × 10^−4^	1.60 ± 0.07	56.04 ± 1.25	0.36	5.96	−96.14	−92.40	0.98
	pH 3.4	2.6 × 10^−5^ ± 2.0 × 10^−5^	2.00 ± 0.18	NaN	0.30	3.25	−82.62	−79.45	0.94
	pH 3.9	4.7 × 10^−6^ ± 2.2 × 10^−5^	1.95 ± 1.06	NaN	0.32	3.68	−78.11	−74.95	0.41

* TLR (time to reach 5-log cell density reduction). NaN not a number.

## Data Availability

The data that supports the findings of this study are available from the corresponding author upon request.

## Supplementary Materials

The following supporting information can be downloaded at: https://www.mdpi.com/article/10.3390/foods15101799/s1, Table S1: Microbial enumeration (Log N, CFU/mL) at refrigeration temperature (5oC), in passion fruit juice with a pH of 2.9, 3.4, and 3.9.
