# Supplementary material for: Harnessing the Natural Acidity of Raw Passion Fruit Juice for Pathogen Inactivation in Developing Countries
Source: Foods. 2026 May 19;15(10):1799. doi: 10.3390/foods15101799 (PMC13205964; doi:10.3390/foods15101799)
Supplement: Supplementary file 1 [file foods-15-01799-s001.zip › foods-4275670-supplementary.pdf]

Supplementary Materials

Table S1: Microbial enumeration (Log N, CFU/mL) at refrigeration temperature (5°C), in passion fruit juice with a pH of 2.9, 3.4, and 3.9.

**Time (h)**	**0**	**4**	**8**	**12**	**24**	**48**	**72**	**96**
Salmonella typhimurium								
pH 2.9	8.21±0.14$_{ab}$	5.04±0.14$_{ef}$	4.50±0.14$_{f}$	2.81±0.14$_{h}$	1.23±0.14$_{i}$	0.70±0.14$_{i}$		
pH 3.4	8.24±0.10$_{a}$		7.33±0.14$_{c}$	6.17±0.14$_{d}$	5.35±0.10$_{e}$	3.67±0.14$_{g}$	3.24±0.14$_{gh}$	1.12±0.16$_{i}$
pH 3.9	8.28±0.14$_{a}$		7.73±0.14$_{abc}$		8.41±0.14$_{a}$	7.51±0.14$_{bc}$	7.46±0.14$_{c}$	6.16±0.14$_{d}$
E. coli O157:H7								
pH 2.9	8.13±0.09$_{a}$	7.61±0.13$_{a}$	6.61±0.13$_{b}$	4.06±0.13$_{d}$	0.78±0.09$_{f}$	0.85±0.13$_{f}$		
pH 3.4	8.09±0.09$_{a}$		8.13±0.09$_{a}$		7.76±0.13$_{a}$	5.85±0.13$_{c}$	3.60±0.13$_{d}$	1.67±0.13$_{e}$
pH 3.9	8.08±0.09$_{a}$	7.85±0.09$_{a}$	7.94±0.13$_{a}$		8.12±0.13$_{a}$	8.19±0.13$_{a}$	7.78±0.13$_{a}$	7.99±0.13$_{a}$
Listeria monocytogenes								
pH 2.9	8.28±0.09$_{ab}$	8.19±0.09$_{ab}$	7.48±0.09$_{de}$	7.42±0.09$_{e}$	6.34±0.09$_{g}$	3.58±0.09$_{i}$	0.98±0.09$_{j}$	
pH 3.4	8.46±0.09$_{a}$		7.91±0.09$_{bcd}$		7.95±0.09$_{bc}$	6.93±0.09$_{f}$	6.05±0.09$_{g}$	4.88±0.09$_{h}$
pH 3.9	8.60±0.09$_{a}$		8.23±0.09$_{ab}$		8.29±0.09$_{ab}$	7.70±0.09$_{cde}$	7.64±0.09$_{cde}$	7.50±0.09$_{cde}$
